# ENSO elicits opposing responses of semi-arid vegetation between Hemispheres

**DOI:** 10.1038/srep42281

**Published:** 2017-02-09

**Authors:** Anzhi Zhang, Gensuo Jia, Howard E. Epstein, Jiangjiang Xia

**Affiliations:** 1 CAS Key Laboratory of Regional Climate-Environment for Temperate East Asia, Institute of Atmospheric Physics, Chinese Academy of Sciences, Beijing 100029, China; 2 Department of Environmental Sciences, University of Virginia, Charlottesville, VA 22904, USA

## Abstract

Semi-arid ecosystems are key contributors to the global carbon cycle and may even dominate the inter-annual variability (IAV) and trends of the land carbon sink, driven largely by the El Niño–Southern Oscillation (ENSO). The linkages between dynamics of semi-arid ecosystems and climate at the hemispheric scale however are not well known. Here, we use satellite data and climate observations from 2000 to 2014 to explore the impacts of ENSO on variability of semi-arid ecosystems, using the Ensemble Empirical Mode Decomposition method. We show that the responses of semi-arid vegetation to ENSO occur in opposite directions, resulting from opposing controls of ENSO on precipitation between the Northern Hemisphere (positively correlated to ENSO) and the Southern Hemisphere (negatively correlated to ENSO). Also, the Southern Hemisphere, with a robust negative coupling of temperature and precipitation anomalies, exhibits stronger and faster responses of semi-arid ecosystems to ENSO than the Northern Hemisphere. Our findings suggest that natural coherent variability in semi-arid ecosystem productivity responded to ENSO in opposite ways between two hemispheres, which may imply potential prediction of global semi-arid ecosystem variability, particularly based on variability in tropical Pacific Sea Surface Temperatures.

The terrestrial ecosystem sink of anthropogenic carbon dioxide (CO_2_) emissions helps mitigate climate change by slowing the increase of atmospheric CO_2_ concentrations^1^. Its large year-to-year variability in responding to climate leads to major uncertainties in estimating the magnitude of this sink^2^. The linkages between terrestrial ecosystems and climate must be better explored to reduce the uncertainties in estimating the land carbon sink, to help fill the gaps in the global CO_2_ budget^2^, and to better understand the impacts of climate variability on inter-annual variations of the global carbon cycle^3^,^4^. Semi-arid ecosystems, with scarcity of water related to low precipitation and high evapotranspiration, are particularly susceptible and vulnerable to climate fluctuation and changes^5^,^6^,^7^, especially drought^8^,^9^,^10^. Meanwhile, global semi-arid areas are projected to expand in the future^11^. Semi-arid ecosystems have been shown to be key contributors to the inter-annual variability of the global (GL) carbon cycle^3^, and may even dominate the variability and trend of the global land carbon sink^4^, which could be attributed to increased ecosystem productivity of Southern Hemisphere (SH) semi-arid vegetation^3^,^4^,^12^; but a comprehensive analysis of the differential responses of the Northern Hemisphere (NH) and SH semi-arid ecosystems is lacking.

Productivity of semi-arid vegetation is mainly constrained by precipitation (P), and further limited by temperature (T) in middle latitudes^6^,^13^. Its inter-annual anomalies are clearly linked to water availability, and are therefore controlled by precipitation and temperature anomalies^4^,^13^,^14^. Global variabilities in precipitation and temperature are to a great extent driven by ENSO^4^,^15^, one of the most prominent year-to-year natural climate phenomena with a global influence that fluctuates between anomalously warm (El Niño) and cold (La Niña) conditions in the tropical Pacific^16^,^17^. However, the heterogeneity of relationships between variability of climate and semi-arid terrestrial ecosystem productivity is not well understood^18^. Here, we investigate the linkages among ENSO, precipitation and temperature anomalies, and vegetation activity of semi-arid areas in both the Northern and Southern Hemispheres (Supplementary Fig. 1), using monthly climate and satellite observations from 2000 to 2014, to improve our understanding of the interactions between climate change and the terrestrial carbon cycle.

To quantify the impacts of ENSO on semi-arid ecosystems, we examined the geographic patterns of trends in Precipitation Condition Index (PCI), Vegetation Condition Index (VCI), precipitation (P), temperature (T), and Normalized Difference Vegetation Index (NDVI) anomalies, as well as their correlations with the Niño3.4 index (Fig.1, see also Supplementary Fig. 2). PCI and VCI are normalized so that they are spatially comparable and independent from absolute values. The climate variables exhibit interesting spatial patterns, i.e., an opposite hemispheric patterns for precipitation - a positive correlation with ENSO in the NH (wetting) and a negative correlation with ENSO in the SH (drying) (Fig. 1a,b,e,f); however, ENSO also has more positive correlation with temperature in both hemispheres (warming), although this is substantially stronger in the SH (Fig. 1c,d). Widespread areas of vegetation production (VCI: 41.5%; NDVI: 42.9%) in the SH show significant negative correlations with ENSO, whereas the NH has larger extents of positive correlations (Fig. 1g,i and Table 1). The NH area with significant greening trends is almost twice as large as the area with significant browning trends for both vegetation indicators (VCI, NDVI); browning areas of the SH are only slightly greater in extent than greening areas (Fig. 1h,j and Table 1).

We find prevailing correspondences of the same sign between precipitation and vegetation productivity for both trends and inter-annual variability (red and blue area in Supplementary Fig. 3), with greater areal percentages of correlation in the SH compared to the NH (Supplementary Table 1). We also see opposite signs in the correlation as well as trends between precipitation and temperature, especially in the SH (Supplementary Fig. 4). The heterogeneous correspondences reveal complex responses of vegetation to changes in climate variables (e.g. those related to ENSO). Also, there may not necessarily be direct causal relationships between the controls on inter-annual variability and those on longer-term trends (Supplementary Fig. 5); in other words, a strong relationship between a climatic variable and inter-annual variation in vegetation productivity does not necessarily suggest that there will be an equally strong relationship between that variable and the trend in vegetation productivity. Whereas vegetation trends have been previously associated with temporal span^19^, numerous other factors can influence long term trends^20^, including the lagged responses of regional vegetation to precipitation and temperature^6^, extreme climate events (e.g. the record greening over the SH in 2011^3^,^15^), human interventions and land cover changes^21^, and the CO_2_ fertilization effect^18^. The areal extent of the greening trend is larger than that of increased precipitation in the NH, but the extent of browning in the SH is equivalent to extent of drying. This could be due to intrinsic characteristics of semi-arid vegetation with regard to capitalization on increased precipitation yet being relatively resistant to drought^18^, in other words, abrupt greening and gradual browning in response to climate variability^7^.

We further investigated the temporal linkages between ENSO and hemispheric semi-arid ecosystem dynamics in terms of inter-annual variability (Supplementary Table 2, and Fig. 6–7), by applying the Ensemble Empirical Mode Decomposition (EEMD) approach^22^ on the regionally averaged time series. Semi-arid ecosystems show coherent variability (Fig. 2a–c) with regard to climate and vegetation. Greater precipitation and increased vegetation productivity were associated with warmer El Niño conditions for the NH, while the SH exhibits the opposite pattern – reduced precipitation and decreased vegetation productivity were linked to warm El Niño^23^. Precipitation and vegetation greenness had significantly positive correlations with ENSO in terms of variability for the NH. Both precipitation and vegetation productivity responded to ENSO with evident time lags; P, PCI, VCI, and NDVI anomalies were lagged by approximately 5, 3, 8, and 7 months in the NH, respectively (Fig. 3 and Supplementary Table 3). In contrast, significantly negative correlations are found between ENSO and these precipitation and vegetation indices in the SH, with lagged responses to ENSO by 2, 2, 3, and 3 months, respectively (Supplementary Fig. 8b). Vegetation variability in semiarid regions are controlled by drought, and generally tend to respond to drought with lagged effects^24^, while vegetation in the mid-latitude NH shows longer responses to drought than in low latitudes where most of the SH occupied^25^. The quicker responses of SH precipitation to ENSO than NH precipitation, indicates differential lagged effects of ENSO impacts on semi-arid ecosystems. The significantly positive correlations between ENSO and temperature found in the SH, occur with maximum correlations of ENSO leading temperature by 2 months (Fig. 3).

Vegetation responded in similar ways to precipitation in both hemispheres, however temperature exhibited the opposite effect on semi-arid vegetation, showing strong, negative controls in the SH, but weaker, positive controls in the NH (Supplementary Fig. 9). Clearly, precipitation plays the dominant role in controlling vegetation variability for semi-arid ecosystems globally^6^,^10^,^13^. Furthermore, ENSO likely resulted in greater water deficit in SH semi-arid ecosystems due to the combined effects of low precipitation and increased heat stress (Supplementary Fig. 10), and further intensified fluctuations of vegetation productivity than in the NH (Fig. 3).

We performed cross correlation analyses between original time series of Nino 3.4 index and land surface variables (P/T anomalies, PCI, VCI and NDVI anomalies) at the pixel scale. We further performed the EEMD analyses for P/T anomalies and PCI in space; however, VCI and NDVI anomalies were excluded as a result of missing data during winter time, especially over mid-latitudes in the NH (see more details in methods and Supplementary Fig. 11).

The NH had greater areas with significant positive correlations for all variables in both original and variability, whereas the SH had areas dominated by significant negative correlations except for temperature (Fig. 4). We find higher percentages of regions with significant correlations (both negative and positive) for EEMD extracted variability than for the original time series for all variables (Fig. 4), as a result of increased correlations in absolute values for the variability, showing a clear spatial pattern of semi-arid ecosystem responses to ENSO (Supplementary Fig. 12–15). Areas with significant cross correlations showed similar characteristics of lag times, generally with the greatest proportions within lag time of 6 months. Furthermore, the areas with significant correlation in the SH were greater than that in the NH, supporting previous findings that indicated more heterogeneous responses of the NH semi-arid ecosystems to ENSO. The PCI had higher correlation and more significant areas compared to the CRU TS3.23 precipitation (positive in the NH, negative in the SH) (Supplementary Fig. 12–13).

Semi-arid regions over most of Australia, Central Asia, and the northwestern USA had quick responses to ENSO in precipitation (time-lag effect is within 1–2 months), while Southern Africa responded to ENSO with the longest time lag of 5–6 months (Fig. 5 and Supplementary Fig. 16). However, variability in temperature showed 6-month delayed responses to ENSO in large areas in Central Asia and North Africa (Fig. 5). The vegetation responses to ENSO with longer lags than precipitation or temperature did (Supplementary Fig. 16), while precipitation and vegetation responded to ENSO faster and more strongly in the SH (Fig. 4), suggesting a chain of ENSO effects on semi-arid ecosystems.

Apparently, ENSO shows key and reverse controls over precipitation and vegetation greenness for hemispheric semi-arid ecosystems, with faster responses over SH (Fig. 3), largely driven by strong and quick atmospheric teleconnections with ENSO in the tropical Pacific bordering areas; while the teleconnections decreased and lagged in areas remote from the Pacific^16^,^26^,^27^, related to different responses of atmospheric circulation and sea surface temperature (SST) to ENSO^28^,^29^. Interestingly, ENSO exhibits the greatest tropical Pacific SST anomalies usually in boreal winter, and has its strongest impacts on semi-arid ecosystems during the local summer time for both hemispheres, suggesting the potential for predicting semi-arid ecosystem productivity using tropical Pacific SST anomalies^16^.

SH semi-arid ecosystems, with strong coupling between precipitation and temperature anomalies, exhibit robust negative and rapid responses to ENSO, however, greening occurred in response to La Niña conditions in recent years. Combined with more heterogeneous connections between ENSO and the NH semi-arid ecosystems, this results in the dominant contribution of SH semi-arid ecosystems to the global carbon cycle^3^,^4^,^15^. However, the contributions of NH semi-arid ecosystems to global carbon cycling may not be ignored. As semi-arid ecosystem productivity is greater during El Niño events and reduced during La Niña for the NH, and the reverse for the SH, the dominant carbon sink will move from one hemisphere to the other as ENSO shifts between warm and cold events. This would lead to substantial hemispheric variability, but potentially more sustained variability of global semi-arid ecosystem. Within the ENSO cycle, SST usually peaks during boreal winter, and sometimes declines rapidly to cold conditions, as what happened between 2009 and 2011. Following high productivity conditions for both hemispheres, the immediate response in the SH to La Niña and the delayed response of the NH to El Niño, combined to yield the extraordinary 2011 land carbon sink (Fig. 3)^3^,^15^.

Our analysis, based on climate data and satellite observations, reveals a natural hemispheric dichotomy for the control of ENSO on the variability of semi-arid vegetation productivity. The stronger and more rapid response to ENSO in the SH, along with robust coupling of precipitation and temperature anomalies, as well as the lagged and contrasting NH responses, combined contribute to the global carbon sink for semi-arid ecosystems. Our findings together with previous studies^3^,^4^,^15^, indicate that semi-arid ecosystems play an important role in estimating and predicting the variability in the global carbon cycle, with robust response to ENSO. Results from this and previous studies may suggest the potential for prediction of semi-arid ecosystem variability, especially based on variability in tropical Pacific SSTs. ENSO is a rather predictable climate phenomenon^12^, and it will likely continue to be the dominant climate signal for inter-annual variability with more extreme El Niño and La Niña events under global warming in the future 17,27,30. More research is needed to investigate the regional differentiation in connections between ENSO characteristics (El Niño or La Niña; frequency or intensity; asymmetry and extremes) and semi-arid ecosystem vegetation productivity, as well as interactions and feedbacks under greenhouse warming scenarios.

## Methods

### Study area

The Global (GL) semi-arid areas investigated in this study are confined as areas with the Humidity Index between 0.2 and 0.5, based on ratio of annual precipitation and potential evapotranspiration within latitude band 50°N–S (http://geodata.grid.unep.ch/)^5^. The semi-arid area covers 13,320,200 km^2^ in NH, approximately 1.86 times as in SH.

### Climate data

The 0.5° resolution monthly mean temperature (TMP) and precipitation total (PRE) datasets from the Climate Research Unit (CRU TS3.23; 1950-2014) are used in this study (http://badc.nerc.ac.uk/browse/badc/cru/data/cru_ts/cru_ts_3.23)^31^. The monthly precipitation (*P*) and temperature (*T*) anomalies were obtained relative to the 1961–1990 climatology. The monthly area average of Sea Surface Temperature (SST) over the Niño3.4 region (the eastern equatorial Pacific of 5°N–5°S and 170°W–120°W) were got from the Climate Prediction Center (CPC: http://www.esrl.noaa.gov/psd/data/correlation/nina34.data) based on the Extended Reconstructed Sea Surface Temperature (ERSST) v3b. The Niño3.4 index of SST anomalies relative to monthly mean of February 2000–August 2014 was used to donate the ENSO properties.

### Remote sensing data and process

We use the globally validated Moderate Resolution Imaging Spectroradiometer (MODIS) products of Normalized Difference Vegetation Index^32^ (NDVI; MOD13C2: monthly, February 2000–August 2014) and land cover classification^33^ with scheme defined by the International Geosphere Biosphere Programme (IGBP) for 2012 (MCD12C1 V051) at 0.05° spatial resolution. Both of the data were obtained from https://lpdaac.usgs.gov/. The MODIS NDVI product has been corrected against atmosphere, clouds and aerosols (more details in the product description of MOD13C2). The NDVI has been extensively used to quantify vegetation productivity and changes over global^13^,^34^,^35^, especially over semi-arid areas, where perform the best based on quality assessment^34^. The anomalies were obtained relative to monthly mean of February 2000–August 2014. The Tropical Rainfall Measuring Mission (TRMM) Multi-satellite Precipitation Analysis (TMPA) 3B43 dataset (version 7) provides validated and high quality quasi-global (50°N–S) monthly precipitation estimates from multiple satellites as well as gauge analyses where feasible at 0.25° spatial resolution^36^, limiting our study area from 50°N to 50°S. The dataset were downloaded from http://mirador.gsfc.nasa.gov/.

The dry and wet spell are inferred by the Precipitation Condition Index (PCI)^37^ and Vegetation Condition Index (VCI)^38^, derived from the following equations based on monthly MODIS NDVI and TRMM precipitation data.


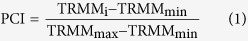



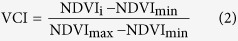


The variables were linearly scaled from 0 to 1 (corresponding to the precipitation/vegetation changes from extremely low to high) for each pixel based on monthly value (TRMM precipitation/NDVI_i_), absolute minimum (TRMM precipitation/NDVI_min_) and maximum values (TRMM precipitation/NDVI_max_) for the same month from February 2000 to August 2014.

### The Ensemble Empirical Mode Decomposition

The variability was extracted using the Ensemble Empirical Mode Decomposition (EEMD) approach^22^, a method based on the original EMD algorithm^39^. The EEMD is a noise-assisted, adaptive, and temporal local data analysis method for analysing any nonlinear and nonstationary time series. In EEMD, time series of regional averaged variables are decomposed into meaningful components on different timescales, while the last component is the nonlinear trend. According to the time scales of each components (Supplementary Table 2; calculated as in reference^40^), we summed the components of time period longer than one year to represent inter-annual variability (IAV). In EEMD calculation, the noise added to data has amplitude of 0.2 standard deviations of the corresponding data and the ensemble number is 1000 as suggested^22^. To better understand the spatial connections between ENSO and variables of semi-arid ecosystems, the EEMD approaches were also performed pixel-by-pixel over global semi-arid regions for CRU TS3.23 monthly precipitation (*P*) and temperature (*T*) anomalies, and TRMM PCI. EEMD decompositions were not applied to MODIS VCI and NDVI anomaly in space, due to large percentage of missing data during winter time especially over NH mid-latitudes (Supplementary Fig. 11). The details of EEMD approach and process can be found in the references. The first and last 6 months of the variability and trends results are excluded to eliminate the minor influence of end effect by EEMD.

### Correlation and trend analysis

This study focuses on changes of semi-arid ecosystems and their relationship with ENSO. The spatial patterns of the Pearson’s correlation coefficient were calculated between Niño3.4 index and land surface variables (*P*/*T* anomalies, PCI, VCI and NDVI anomalies); and the linear trend was estimated using ordinary least squares method for the same time period. Furthermore, lagged correlations were calculated between variables for original and EEMD extracted variability at pixel scale and regional averaged time series. The time period of correlation was February 2000–August 2014 for all variables. The significance levels of linear correlation and trend (*p* value) were calculated using a two-tailed Student’s t-test and a non-parametric Mann-Kendall test, respectively. Finally, the statistics of linear correlation and trend for the spatial distributions and regional averages were summarized to qualify their concurrent and lagged association.

## Additional Information

**How to cite this article:** Zhang, A. *et al*. ENSO elicits opposing responses of semi-arid vegetation between Hemispheres. *Sci. Rep.* **7**, 42281; doi: 10.1038/srep42281 (2017).

**Publisher's note:** Springer Nature remains neutral with regard to jurisdictional claims in published maps and institutional affiliations.

## Supplementary Material

Supplementary Information

## Figures and Tables

**Figure 1:**
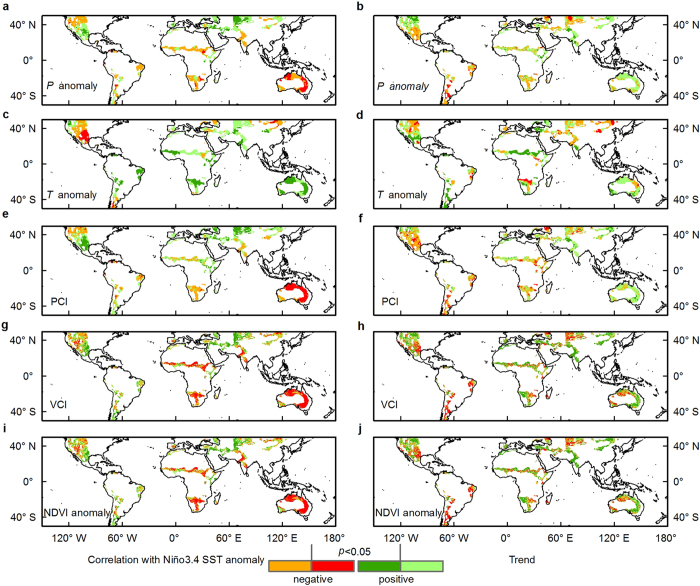
Spatial patterns of correlations and linear trends over global semi-arid areas from 2000 to 2014. (**a**,**c**,**e**,**g**,**i**) The correlations between monthly mean Niño3.4 index and Climate Research Unit (CRU TS3.23) precipitation anomaly (*P* anomaly, **a**), temperature anomaly (*T* anomaly, **c**), TRMM precipitation condition index (PCI, **e**), MODIS vegetation condition index (VCI, **g**) and NDVI anomaly (**i**). (**b**,**d**,**f**,**h**,**j**) The linear trends of *P* anomaly (**b**; mm yr^−1^), *T* anomaly (**d**; °C yr^−1^), TRMM PCI (**f**; yr^−1^), MODIS VCI (**h**; yr^−1^) and NDVI anomaly (**j**; yr^−1^) are shown in the right panels. The PCI, VCI and NDVI anomaly are unitless. Statistically significant of trends and correlations (values lower or greater than 0.1484) at the 95% significance level (P < 0.05) are indicated in red or dark green color. Note that the values are classified into two categories as negative and positive. The maps were created by the ArcMap 10.1 (http://www.esri.com/software/arcgis/arcgis-for-desktop).

**Figure 2:**
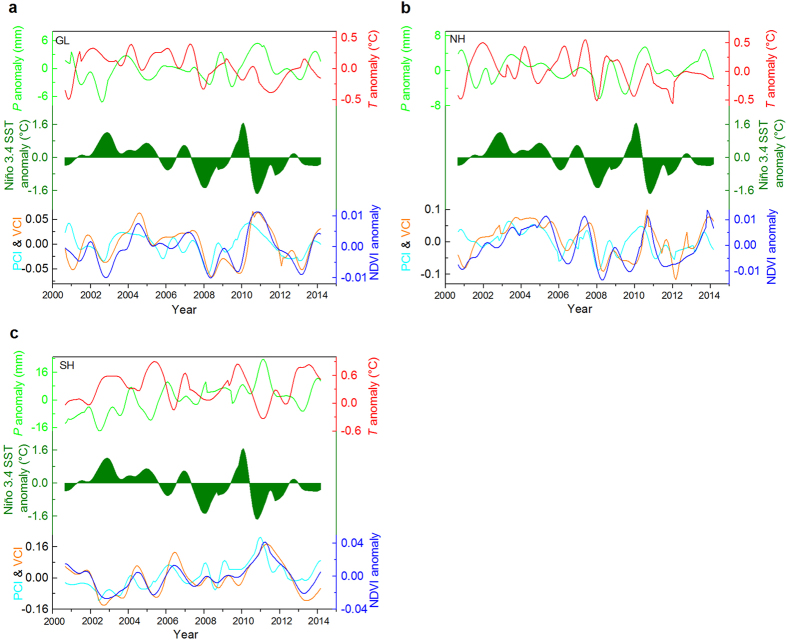
Variability extracted using the Ensemble Empirical Mode Decomposition (EEMD) method across semi-arid areas from 2000 to 2014. (**a**), Temporal evolution of variability for monthly mean Niño3.4 index (olive shading), Climate Research Unit (CRU TS3.23) precipitation anomaly (*P* anomaly, green line), temperature anomaly (*T* anomaly, red line), TRMM precipitation condition index (PCI, cyan line), MODIS vegetation condition index (VCI, orange line) and NDVI anomaly (blue line) over Global (GL) semi-arid areas. (**b**,**c**), As in **a**, but for the Northern Hemisphere (NH) and Southern Hemisphere (SH) semi-arid areas. The PCI, VCI and NDVI anomaly are unitless.

**Figure 3:**
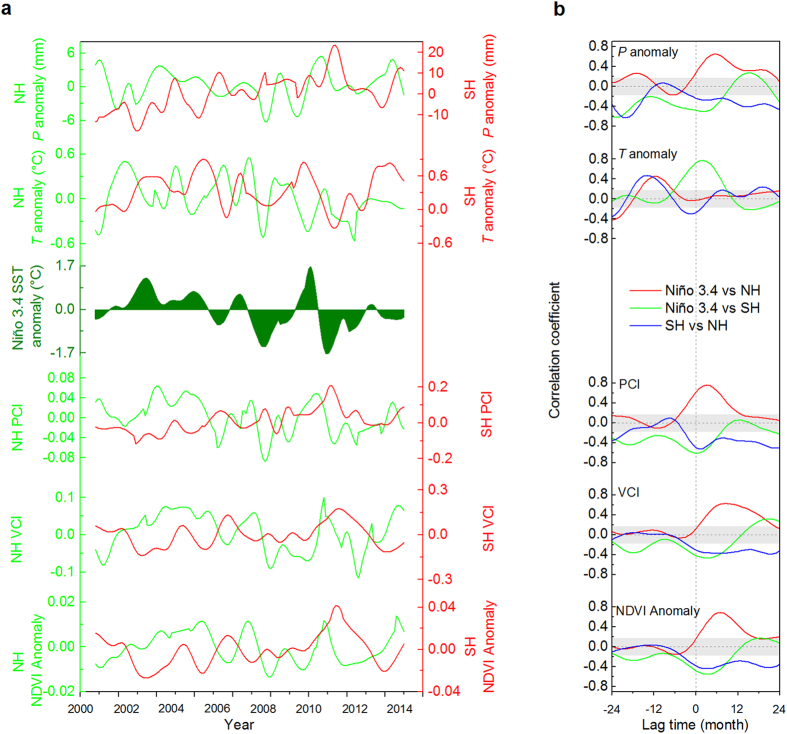
The Ensemble Empirical Mode Decomposition (EEMD) extracted variability of the NH and SH semi-arid areas’ CRU TS3.23 *P* anomaly, *T* anomaly, TRMM PCI, MODIS VCI and NDVI anomaly (**a**), and their cross correlations with Niño3.4 index (**b**). Positive lags mean variables lagging of NH to Niño3.4 (red lines), SH to Niño3.4 (green lines), and SH to NH (blue lines), respectively. Significance levels (*p* > 0.05) are shown in grey shading.

**Figure 4:**
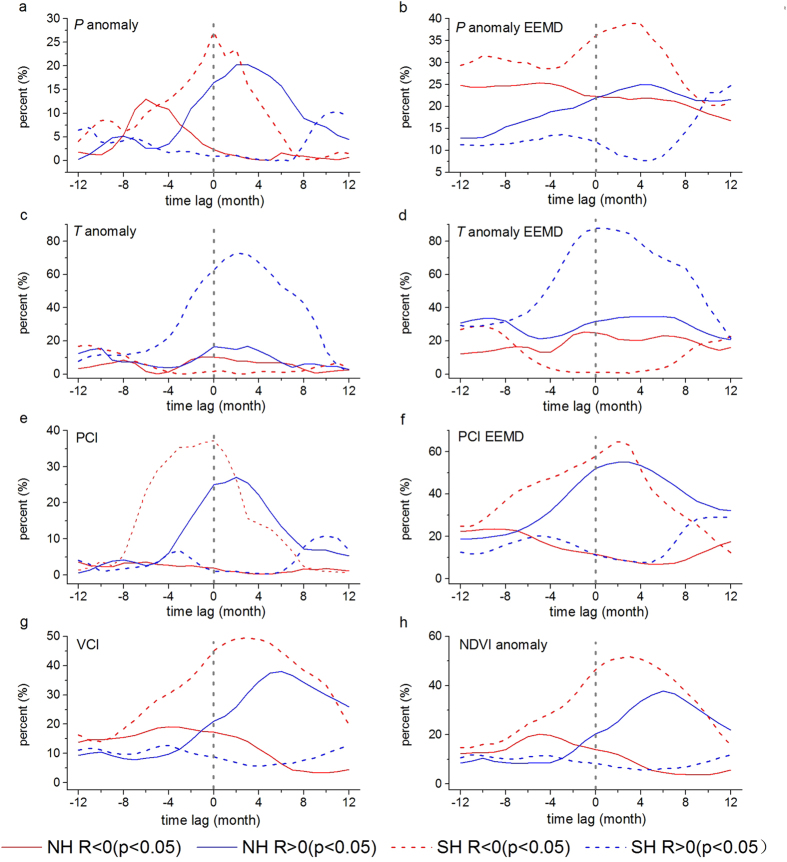
Statistic summary for percentages (%) of pixels with statistically significant (*p* < 0.05) cross correlations (R) between Niño3.4 index and variables for Original time-series and EEMD extracted Variability over NH and SH semi-arid areas. (**a**,**c**,**e**,**g**,**h**) The areal percentages between original monthly mean Niño3.4 index and Climate Research Unit (CRU TS3.23) precipitation anomaly (*P* anomaly, **a**), temperature anomaly (*T* anomaly, **c**), TRMM precipitation condition index (PCI, **e**), MODIS vegetation condition index (VCI, **g**) and NDVI anomaly (**h**). (**b**,**d**,**f**) The statistics between EEMD extracted Variability of monthly mean Niño3.4 index and Climate Research Unit (CRU TS3.23) precipitation anomaly (*P* anomaly EEMD, **b**), temperature anomaly (*T* anomaly EEMD, **d**), TRMM precipitation condition index (PCI EEMD, **f**). Positive lags mean that the Niño3.4 SST anomaly is leading.

**Figure 5:**
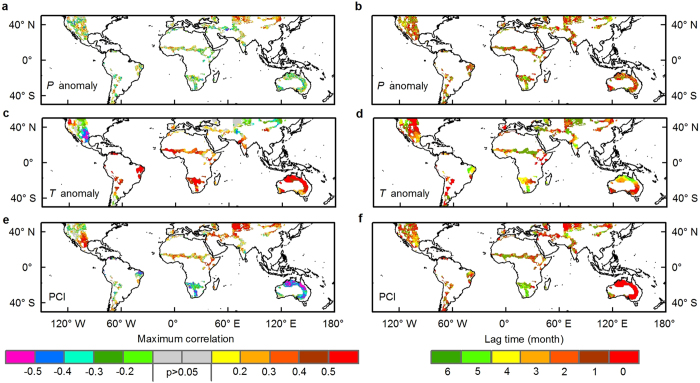
Spatial patterns of maximum cross correlations (R) and time lags between EEMD extracted variability of Niño3.4 index and variables over global semi-arid areas from 2000 to 2014. (**a**,**c**,**e**) The maximum correlations between monthly mean Niño3.4 index and Climate Research Unit (CRU TS3.23) precipitation anomaly (*P* anomaly, **a**), temperature anomaly (*T* anomaly, **c**), TRMM precipitation condition index (PCI, **e**). (**b**,**d**,**f**) The corresponding time lags of *P* anomaly (**b**), *T* anomaly (**d**), TRMM PCI (**f**) are shown in the right panels. Significance levels (*p* > 0.05) are shown in grey shading. Note that the values are calculated from lag times from 0 to 6 months. Time lags mean variables lagging of Niño3.4. The maps were created by the ArcMap 10.1 (http://www.esri.com/software/arcgis/arcgis-for-desktop).

**Table 1:** Statistic summary of correlations between monthly Niño3.4 index and variables (a), and linear trends (b) for semi-arid areas.

(a) Correlation (R)	GL		NH		SH	
variable	R < 0	R > 0	R < 0	R > 0	R < 0	R > 0
*P* anomaly	10.3	11.5	2.3	16.5	27.2	1
*T* anomaly	7.4	31.2	10.1	16.4	1.8	62.6
PCI	12.6	15.4	1.6	71.3	35.1	0.7
VCI	23.8	16.1	15.2	19.7	41.5	8.7
NDVI anomaly	22.4	15.7	12.4	19.2	42.9	8.4
**(b) Trend (S)**	**GL**		**NH**		**SH**	
**variable**	**S < 0**	**S < 0**	**S < 0**	**S < 0**	**S < 0**	**S < 0**
*P* anomaly	5.2	7.4	3.4	10.5	8.2	1.1
*T* anomaly	7.9	23.8	8	22	7.5	25.4
PCI	10.0	8.0	8.9	9.7	12.3	4.5
VCI	21.0	31.7	17.3	33.8	28.6	24.2
NDVI anomaly	20.9	31.4	17.4	34.5	27.9	25.0

The percentages (%) of pixels with statistically significant (*p* < 0.05) negative and positive correlations/trends are listed.
