# Supplementary material for: ENSO elicits opposing responses of semi-arid vegetation between Hemispheres
Source: Sci Rep. 2017 Feb 9;7:42281. doi: 10.1038/srep42281 (PMC5299407; doi:10.1038/srep42281)
Supplement: Supplementary Information [file srep42281-s1.pdf]

SUPPLEMENTARY INFORMATION

ENSO elicits opposing responses of semi-arid vegetation between Hemispheres

Anzhi Zhang[1], Gensuo Jia[1,*], Howard E. Epstein[2], Jiangjiang Xia[1]

1. Key Laboratory of Regional Climate-Environment for East Asia (TEA), Institute of Atmospheric Physics, Chinese Academy of Sciences, Beijing 100029, China
2. Department of Environmental Sciences, University of Virginia, Charlottesville, VA 22904, USA

*Corresponding author: Gensuo Jia (jiong@tea.ac.cn)

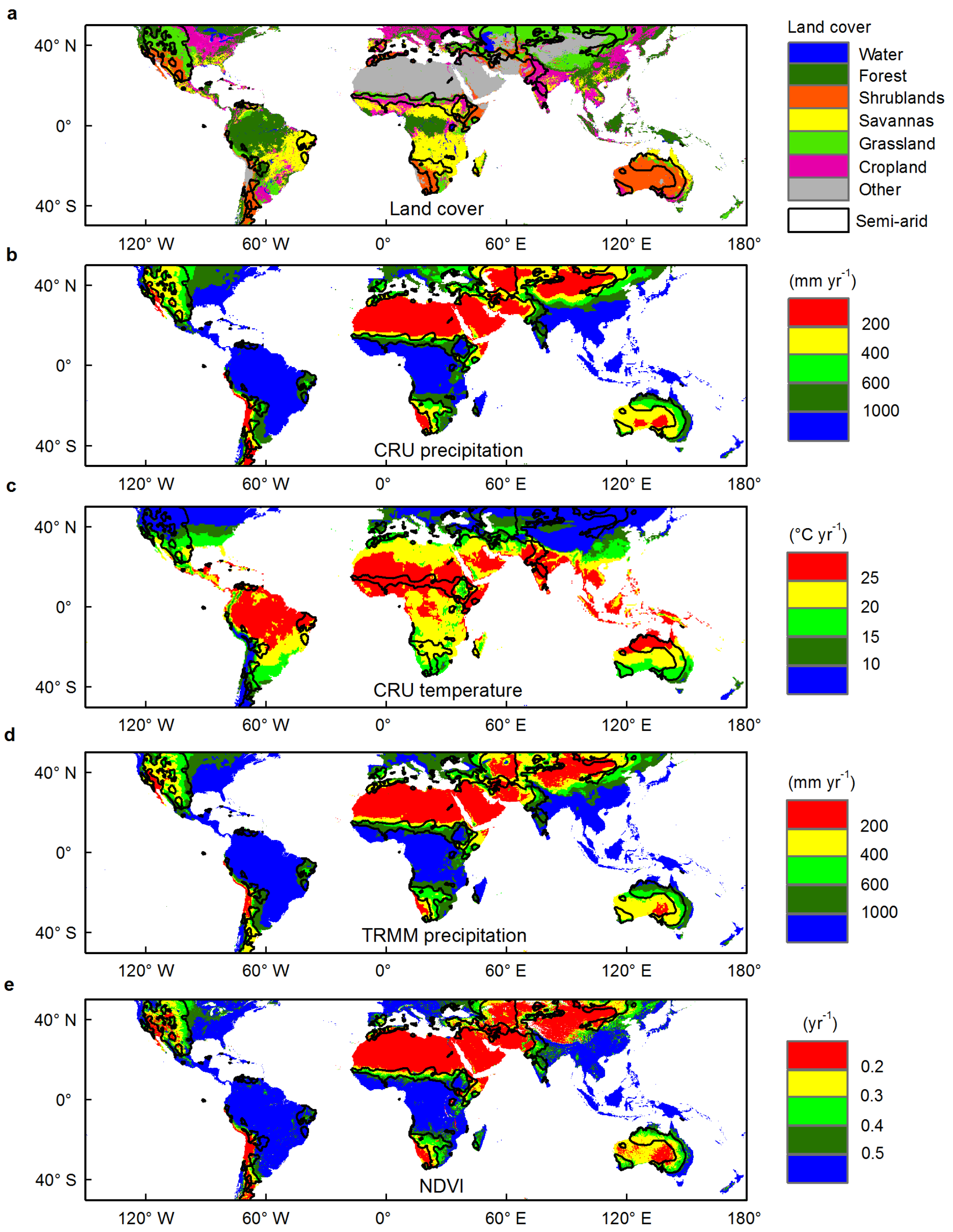

Supplementary Figure 1

Quasi-global land cover classes and characteristics in climatologies. a, Major land cover classes derived from the 2012 MODIS MCD12C1 product with classification scheme of IGBP. **b-e**, Climatologies for annual mean CRU TS3.23 precipitation (**b**), temperature (**c**), TRMM precipitation (**d**), and MODIS NDVI (**e**). The reference period for CRU TS3.23 precipitation and temperature is 1961-1990, where 2000-2014 is used for TRMM precipitation and MODIS NDVI. The maps were created by the ArcMap 10.1 (http://www.esri.com/software/arcgis/arcgis-for-desktop).

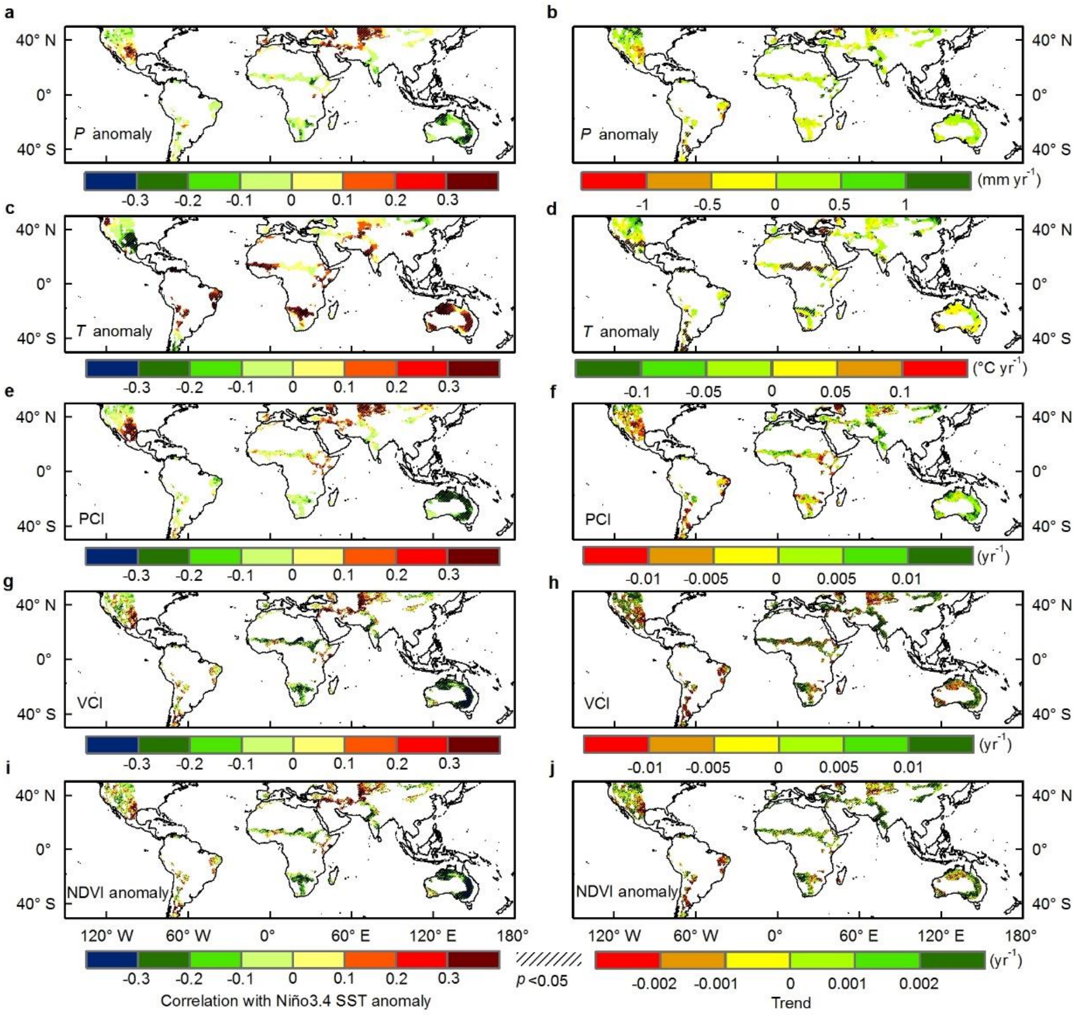

Supplementary Figure 2

Spatial patterns of correlations and linear trends over global semi-arid areas from 2000 to 2014. a,**c**,**e**,**g**,**i**, The correlations between monthly mean Niño3.4 region (5°N–5°S, 170°–120°W) sea surface temperature (SST) anomaly (Niño3.4 index) and Climate Research Unit (CRU TS3.23) precipitation anomaly (*P* anomaly; **a**), temperature anomaly (*T* anomaly; **c**), TRMM precipitation condition index (PCI; **e**), MODIS vegetation condition index (VCI; **g**) and NDVI anomaly (**i**). **b**,**d**,**f**,**h**,**j**, The linear trends of *P* anomaly (**b**; mm yr^{-1}), *T* anomaly (**d**; °C yr^{-1}), TRMM PCI (**f**; yr^{-1}), MODIS VCI (**h**; yr^{-1}) and NDVI anomaly (j; yr^{-1}) are shown in the right panels. The PCI, VCI and NDVI anomaly are unitless. Statistically significant of trends and correlations at the 95% level ($P < 0.05$) are indicated by hatching. The anomalies of precipitation and temperature (NDVI anomaly) are with respect to 1961-1990 (February 2000–August 2014) monthly means. The TRMM PCI, MODIS VCI, NDVI anomaly, CRU TS3.23 precipitation and temperature are derived from monthly values from February 2000 to August 2014. The maps were created by the ArcMap 10.1 (http://www.esri.com/software/arcgis/arcgis-for-desktop).

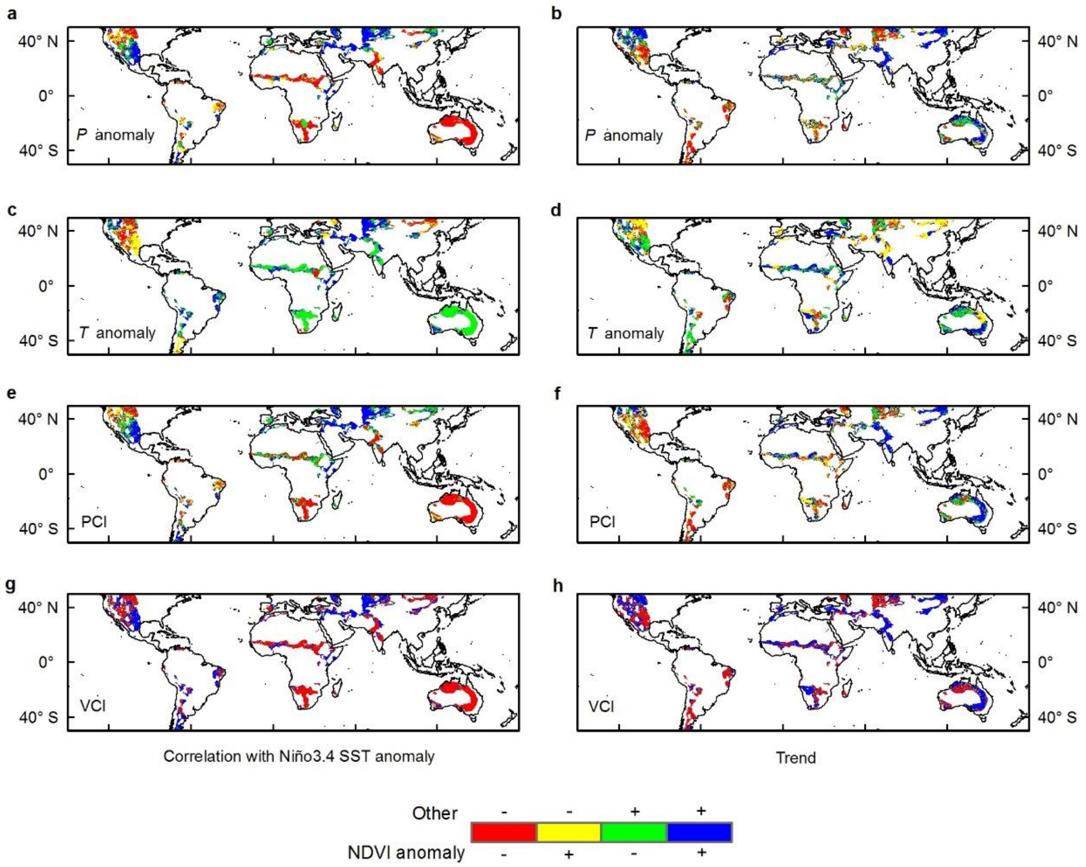

Supplementary Figure 3

Spatial patterns of correspondence in trends and correlations between NDVI anomaly and other variables across global semi-arid areas from 2000 to 2014. **a**,**c**,**e**,**g**, The combinations of correlations (between variables and Niño3.4 index) are given in left panels between NDVI anomaly and CRU TS3.23 *P* anomaly (**a**), *T* anomaly (**c**), TRMM PCI (**e**), MODIS VCI (**g**), respectively. **b**,**d**,**f**,**h**, The linear trends differences are shown in the right panels between NDVI anomaly and CRU TS3.23 *P* anomaly (**b**), *T* anomaly (**d**), TRMM PCI (**f**), MODIS VCI (**h**), respectively. Colors represent different combinations; for example, pixels in yellow are with negative value of other variables (*P* anomaly, *T* anomaly, TRMM PCI, MODIS VCI) and positive value of NDVI anomaly for correlation (**a**,**c**,**e**,**g**) and trend (**b**,**d**,**f**,**h**), respectively. Colors in red and blue show the same negative or positive sign for trends or correlations between NDVI anomaly and other variables, where yellow and green colors exhibit opposite directions. The data are resampled to 0.05° spatial resolution to be the same as MODIS NDVI using a nearest neighbor resampling approach. The maps were created by the ArcMap 10.1 (http://www.esri.com/software/arcgis/arcgis-for-desktop).

	Correlation						Trend					
Other	+	+	-	-	+ -	+ -	+	+	-	-	+ -	+ -
NDVI anomaly	+	-	+	-	+ -	- +	+	-	+	-	+ -	- +
a) GL												
P anomaly	30.4%	15.6%	15.0%	39%	69.4%	30.6%	37.8%	17.9%	18.5%	25.8%	63.6%	36.4%
T anomaly	30.3%	40.3%	15%	14.4%	44.7%	55.3%	28.4%	29.4%	28%	14.2%	42.6%	57.4%
PCI	33.8%	21.4%	11.5%	33.3%	67.1%	32.9%	35.2%	16.1%	21.1%	27.6%	62.8%	37.2%
VCI	41.6%	3.3%	3.7%	51.4%	93.0%	7.0%	53.1%	3.9%	3.2%	39.8%	92.9%	7.1%
b) NH												
P anomaly	39.9%	19.2%	13.1%	27.8%	67.7%	32.3%	42.9%	17.4%	17.7%	22%	64.9%	35.1%
T anomaly	34.1%	26.9%	18.9%	20.1%	54.2%	45.8%	27.5%	25.5%	33.1%	13.9%	41.4%	58.9%
PCI	43.0%	28.3%	9.9%	18.8%	61.8%	38.2%	37.6%	14.5%	23.1%	24.8%	62.4%	37.6%
VCI	48.6%	3.5%	4.4%	43.5%	92.1%	7.9%	57.5%	3.9%	3.2%	35.4%	92.9%	7.1%
c) SH												
P anomaly	10.7%	8%	18.7%	62.6%	73.3%	26.7%	27.2%	18.8%	20%	34%	61.2%	38.8%
T anomaly	22.4%	67.6%	6.9%	3.1%	25.5%	74.5%	30%	37.2%	17.2%	15.6%	45.6%	54.4%
PCI	14.8%	7.2%	14.8%	63.2%	78.0%	22.0%	30.4%	19.4%	17.0%	33.2%	63.6%	36.4%
VCI	27.2%	2.7%	2.3%	67.8%	95.0%	5.0%	44.0%	3.8%	3.4%	48.8%	92.8%	7.2%

Supplementary Table 1

Statistics of the percentages of pixels in correspondence for correlations (variables vs Niño3.4 index) and trend between NDVI anomaly and other variables across semi-arid areas. Signs represent different combinations; for example, percentages of pixels with other (+) and NDVI anomaly (+) correspond to both positive correlation (trend).

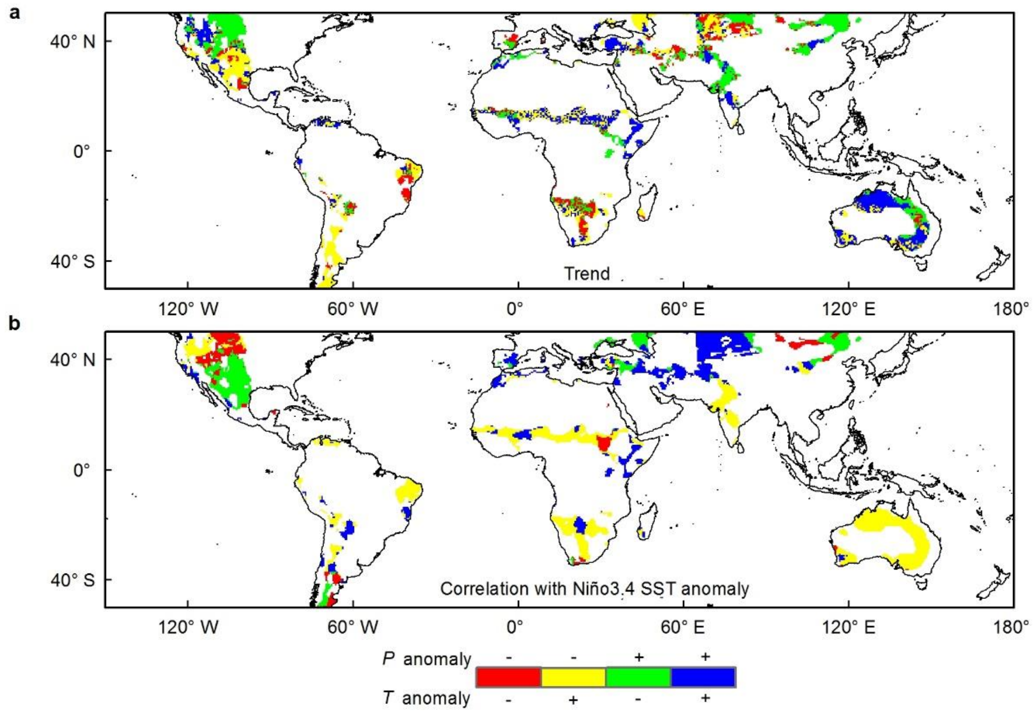

Supplementary Figure 4

Spatial patterns of coupling between CRU TS3.23 ***P* anomaly and *T* anomaly. a**, Trend. **b**, Correlation with Niño3.4 index. Colors represent different combinations; for example, pixels in yellow are with negative value of *P* anomaly and positive value of *T* anomaly for trend (**a**) and correlation (**b**), respectively. The maps were created by the ArcMap 10.1 (http://www.esri.com/software/arcgis/arcgis-for-desktop).

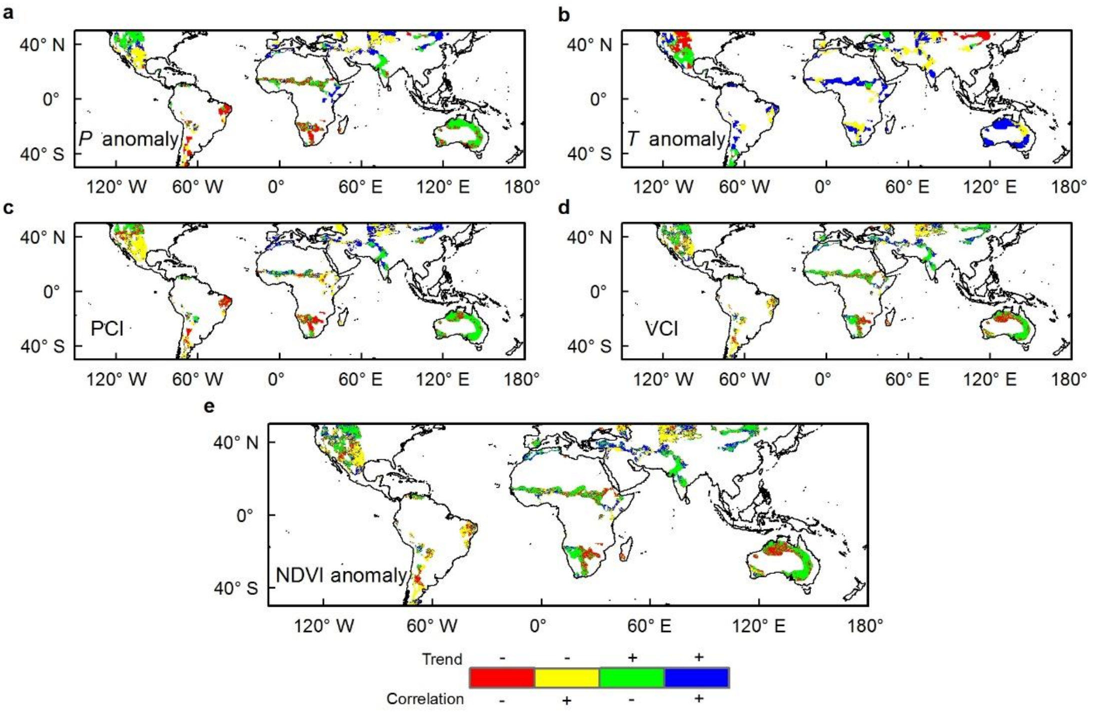

Supplementary Figure 5

Spatial patterns of relationship between correlations and trends over global semi-arid areas from 2000 to 2014. a-e, The combinations for CRU TS3.23 *P* anomaly (**a**), *T* anomaly (**b**), TRMM PCI (**c**), MODIS VCI (**d**) and NDVI anomaly (**e**) are shown in different colors. For example, pixels in red are with trend (-) and correlation (+) correspond to negative (-) trend together with positive (+) correlation with Niño3.4 region SST anomaly. The maps were created by the ArcMap 10.1 (http://www.esri.com/software/arcgis/arcgis-for-desktop).

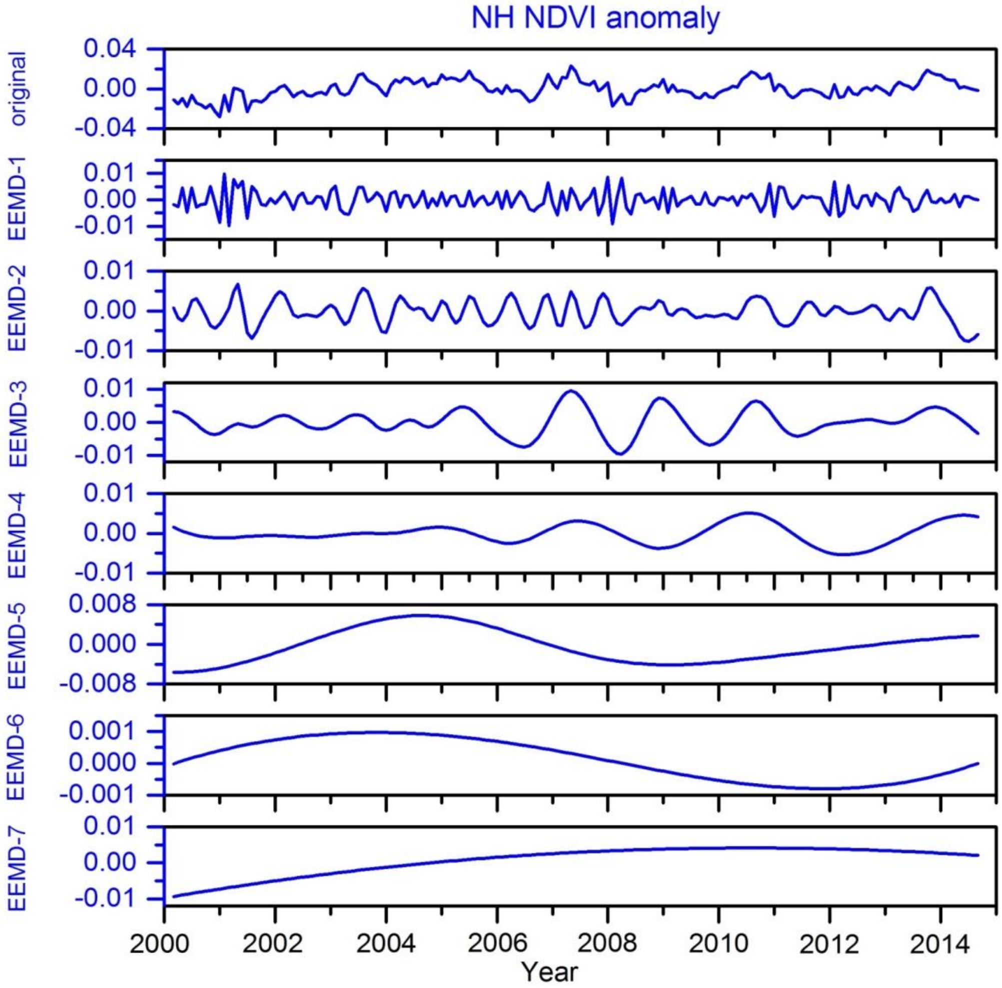

Supplementary Figure 6

Example of EEMD decompositions for NDVI anomaly of the Northern Hemisphere (NH) semi-arid region from February 2000 to August 2014. The top panel (original) stands for the raw time series and the individual components after decomposition are displayed in the next 7 panels (EEMD 1–7), where EEMD–7 is the nonlinear trend extracted by the EEMD method.

	EEMD 1	EEMD 2	EEMD 3	EEMD 4	EEMD 5	EEMD 6
Niño3.4 SST anomaly	0.35	0.78	1.86	3.47	10.62	13.85
P anomaly_GL	0.26	0.57	1.12	2.83	4.52	10.81
P anomaly_NH	0.28	0.57	1.16	2.79	6.10	8.70
P anomaly_SH	0.28	0.63	1.19	2.48	5.04	14.33
T anomaly_GL	0.27	0.55	1.10	1.91	3.68	13.68
T anomaly_NH	0.28	0.57	1.08	2.33	5.36	13.49
T anomaly_SH	0.27	0.60	1.43	2.87	5.53	13.84
PCI_GL	0.28	0.56	1.11	3.02	6.44	8.60
PCI_NH	0.26	0.59	1.10	2.73	5.82	12.16
PCI_SH	0.27	0.57	1.04	2.24	5.96	13.93
VCI_GL	0.32	0.76	1.57	3.58	7.84	13.32
VCI_NH	0.33	0.76	1.53	2.87	8.72	12.78
VCI_SH	0.34	0.66	2.21	4.20	6.81	12.49
NDVI anomaly_GL	0.30	0.65	1.64	3.42	6.83	14.84
NDVI anomaly_NH	0.30	0.72	1.62	3.11	10.22	13.93
NDVI anomaly_SH	0.35	0.77	2.36	4.11	10.99	13.55

Supplementary Table 2

Periods of EEMD decomposition components. The mean periods (year) of various timescale components for monthly Niño3.4 region (5°N–5°S, 170°–120°W) sea surface temperature (SST) anomaly (Niño3.4 index), Global (GL), the Northern Hemisphere (NH) and the Southern Hemisphere (SH) semi-arid area averaged CRU TS3.23 precipitation anomaly (*P* anomaly), temperature anomaly (*T* anomaly), TRMM precipitation condition index (PCI), MODIS vegetation condition index (VCI) and NDVI anomaly derived by the EEMD method, respectively. The green shaded components and part of the components in red color are summed to represent inter-annual variability (time period longer than one year).

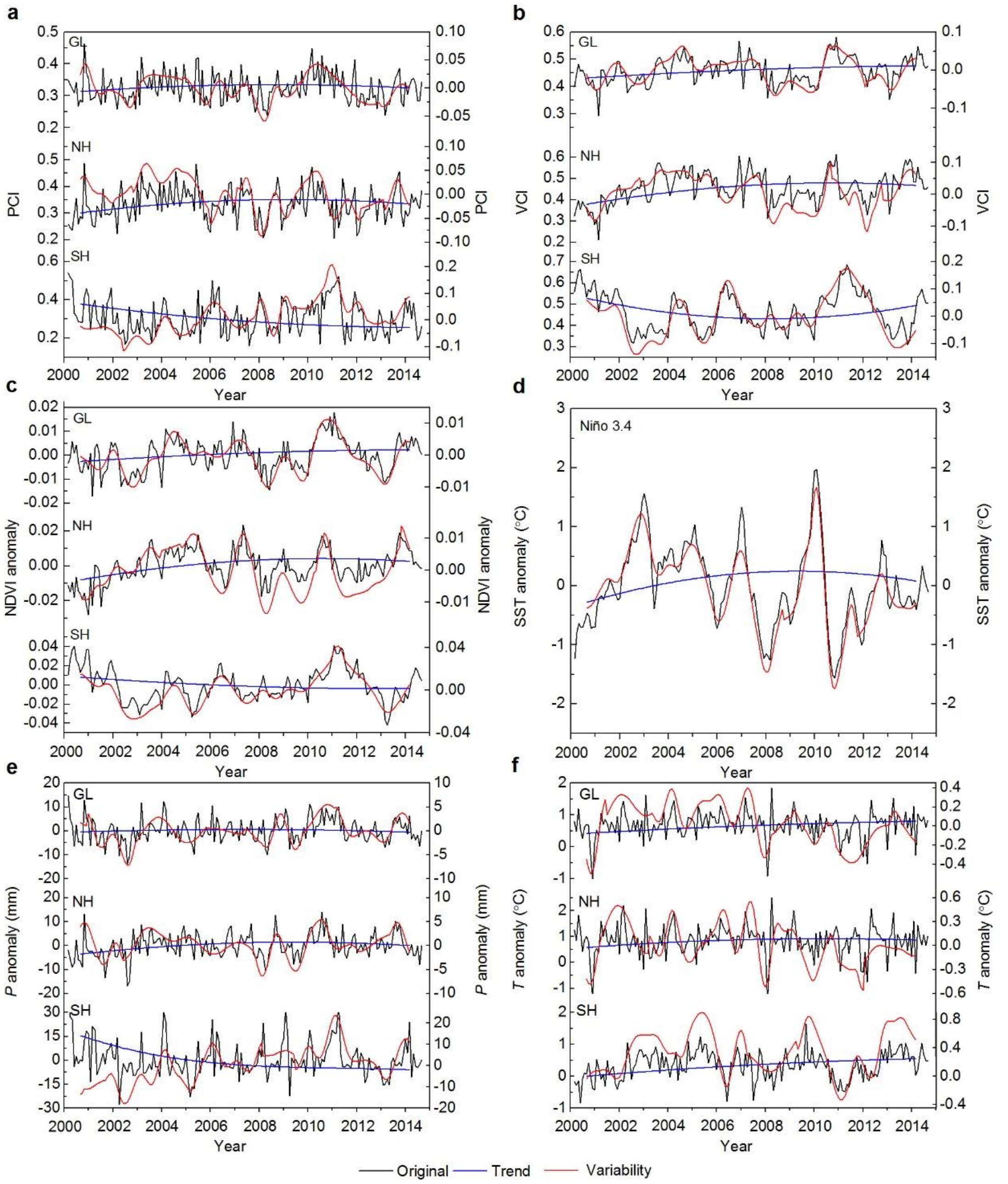

Supplementary Figure 7

Time series for monthly mean climate and remote sensing indices over semi-arid areas. a-**f**, For TRMM precipitation condition index (PCI; **a**), MODIS vegetation condition index (VCI; **b**), MODIS NDVI anomaly (**c**), Niño3.4 region SST anomaly (**d**), CRU TS3.23 precipitation anomaly (*P* anomaly; **e**) and temperature anomaly (*T* anomaly; **f**) of GL, the NH, and SH semi-arid area. In each panel, the black lines represent raw time-series; the blue lines indicate the trend extracted by the EEMD method from February 2000 to August 2014. And the red lines are the variability extracted by the EEMD method. Note that the variability (red line) belongs to right axis of each panel. The EEMD time series show substantial variability and clear nonlinear trends.

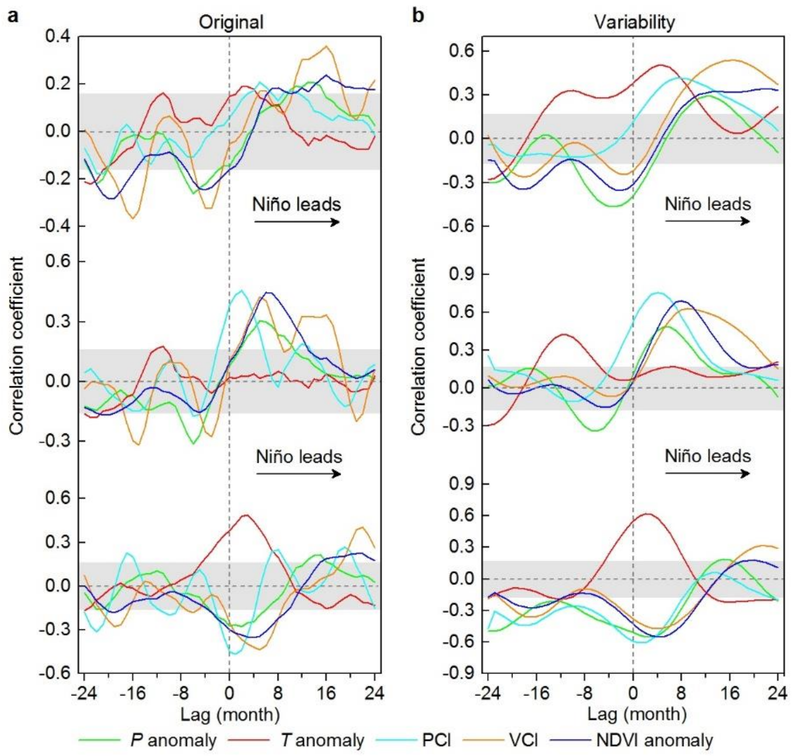

Supplementary Figure 8

Cross correlations between Niño3.4 index and CRU TS3.23 *P* anomaly (green), *T* anomaly (red), TRMM PCI (cyan), MODIS VCI (orange) and NDVI anomaly (blue) for Original time-series (a) and EEMD extracted Variability (b) over GL, NH and SH semi-arid areas. Positive lags mean that the Niño3.4 SST anomaly is leading. Significance levels (p>0.05) are shown in grey shading. Note that similar lagged correlations were found in the Original and Variability time-series; therefore, unnatural connections are not artificially introduced by the EEMD method.

		Original		Variability	
		R (lag=0)	|R|max (lag)	R (lag=0)	|R|max (lag)
GL	*P* anomaly	-0.146*	-0.261(-6)	-0.388	-0.464(-4)
	T anomaly	0.149*	-0.221(-23)	0.384	0.505(5)
	PCI	0.080*	0.242(5)	0.177	0.416(7)
	VCI	-0.175	-0.326(16)	-0.176	0.540(15)
	NDVI anomaly	-0.195	-0.317(-19)	-0.261	-0.356(-3)
NH	*P* anomaly	0.081*	-0.314(-6)	0.114*	0.484(5)
	T anomaly	0.021*	0.176(-11)	0.077*	0.423(-11)
	PCI	0.333	0.408(3)	0.618	0.760(3)
	VCI	0.043*	0.489(7)	0.119*	0.630(8)
	NDVI anomaly	0.115*	0.559(6)	0.160*	0.692(7)
SH	*P* anomaly	-0.272	-0.278(2)	-0.510	-0.552(2)
	T anomaly	0.383	0.487(3)	0.551	0.610(2)
	PCI	-0.340	-0.340(0)	-0.608	-0.608(0)
	VCI	-0.353	-0.425(5)	-0.417	-0.471(3)
	NDVI anomaly	-0.361	-0.452(4)	-0.479	-0.553(3)

* p>0.05

Supplementary Table 3

Summary of cross correlations (R) between Niño3.4 index and CRU TS3.23 *P* anomaly, *T* anomaly, TRMM PCI, MODIS VCI and NDVI anomaly for Original time-series and EEMD extracted Variability over GL, NH and SH semi-arid areas. The Pearson correlation coefficients (R) of no lag (lag=0) and maximum absolute value (|R|max) with lag months given in brackets are shown in different columns. Positive lags mean that the Niño3.4 SST anomaly is leading. Statistically significant correlations at the 95% level are indicated without asterisk (*).

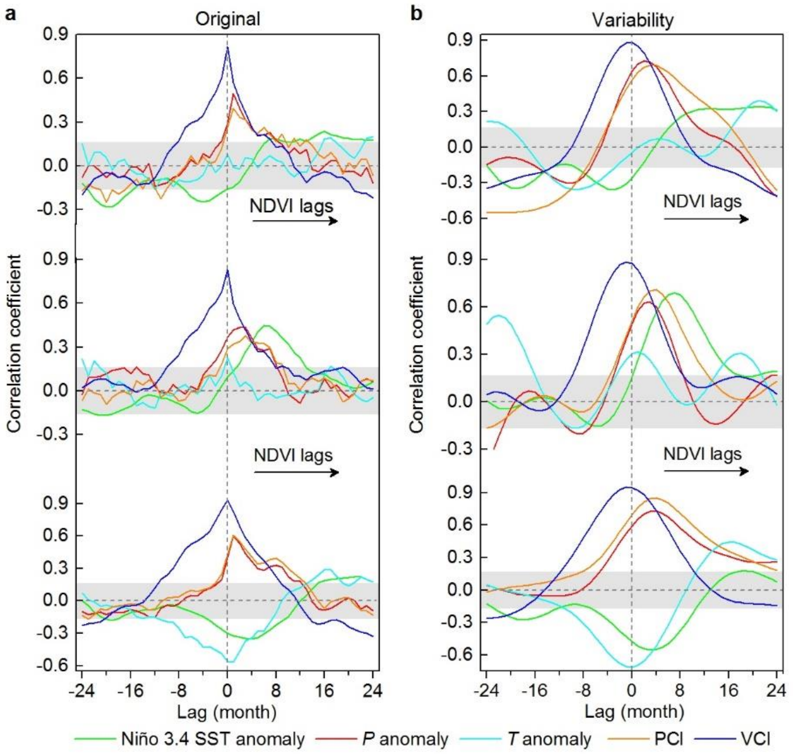

Supplementary Figure 9

Cross correlations between NDVI anomaly and Niño3.4 index (green), CRU TS3.23 *P* anomaly (red), *T* anomaly (cyan), TRMM PCI (orange), MODIS VCI (blue) for Original time-series (a) and EEMD extracted Variability (b) over GL, NH and SH semi-arid areas from 2000 to 2014. Positive lags mean that the NDVI anomaly is lagging. Significance levels (p>0.05) are shown in grey shading. Note that similar lagged correlations were found in the Original and Variability time-series; therefore, unnatural connections are not artificially introduced by the EEMD method.

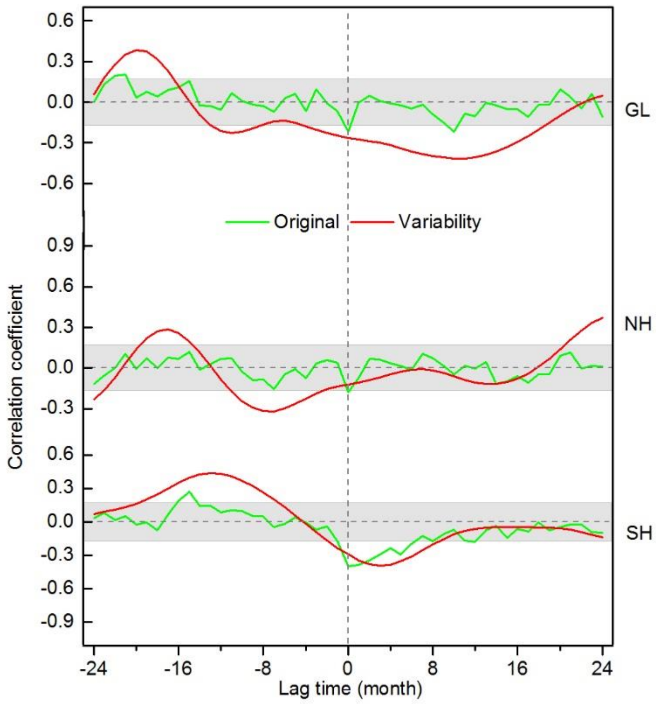

Supplementary Figure 10

Cross correlations of Original time series (green) and EEMD extracted Variability (red) between CRU TS3.23 *P* anomaly and *T* anomaly over GL, NH and SH semi-arid areas from 2000 to 2014. Positive lags mean that the *P* anomaly is leading. Significance levels (p>0.05) are shown in grey shading.

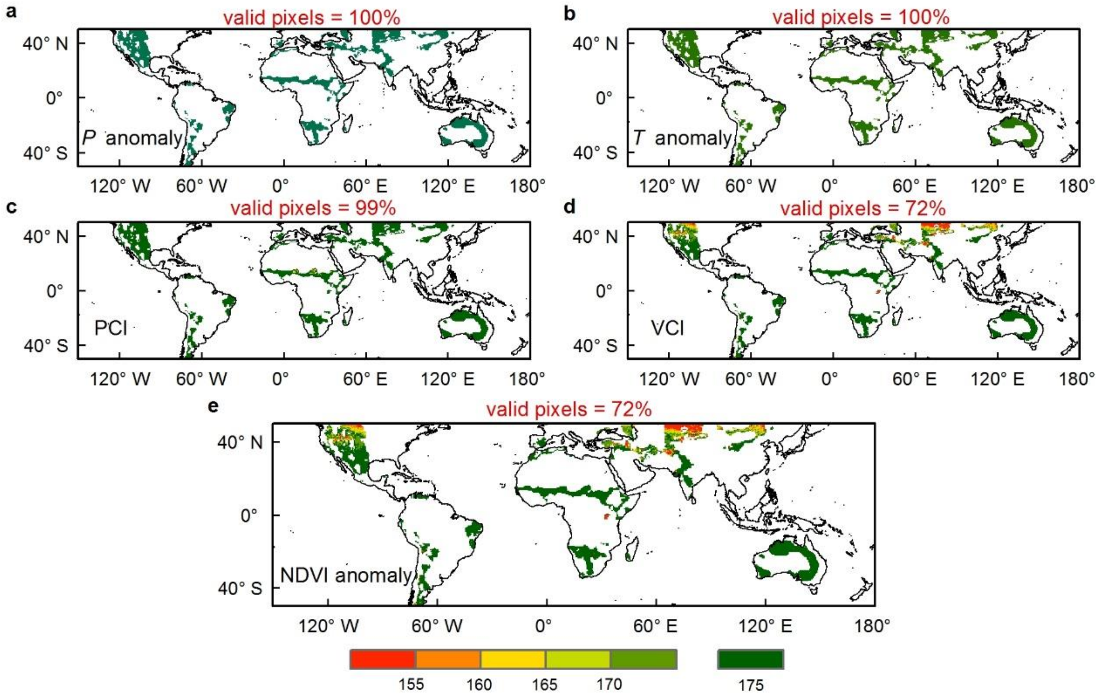

Supplementary Figure 11

Valid numbers for original time series of *P* anomaly (a), *T* anomaly (b), PCI (c), VCI (d), and NDVI anomaly (e) at each pixel over global semi-arid areas from 2000 to 2014. Note that the maximum number is 175 if the time series have no missing data. The percentages of pixels with maximum number for global semi-arid area are indicated as valid pixels in each panel. The maps were created by the ArcMap 10.1 (http://www.esri.com/software/arcgis/arcgis-for-desktop).

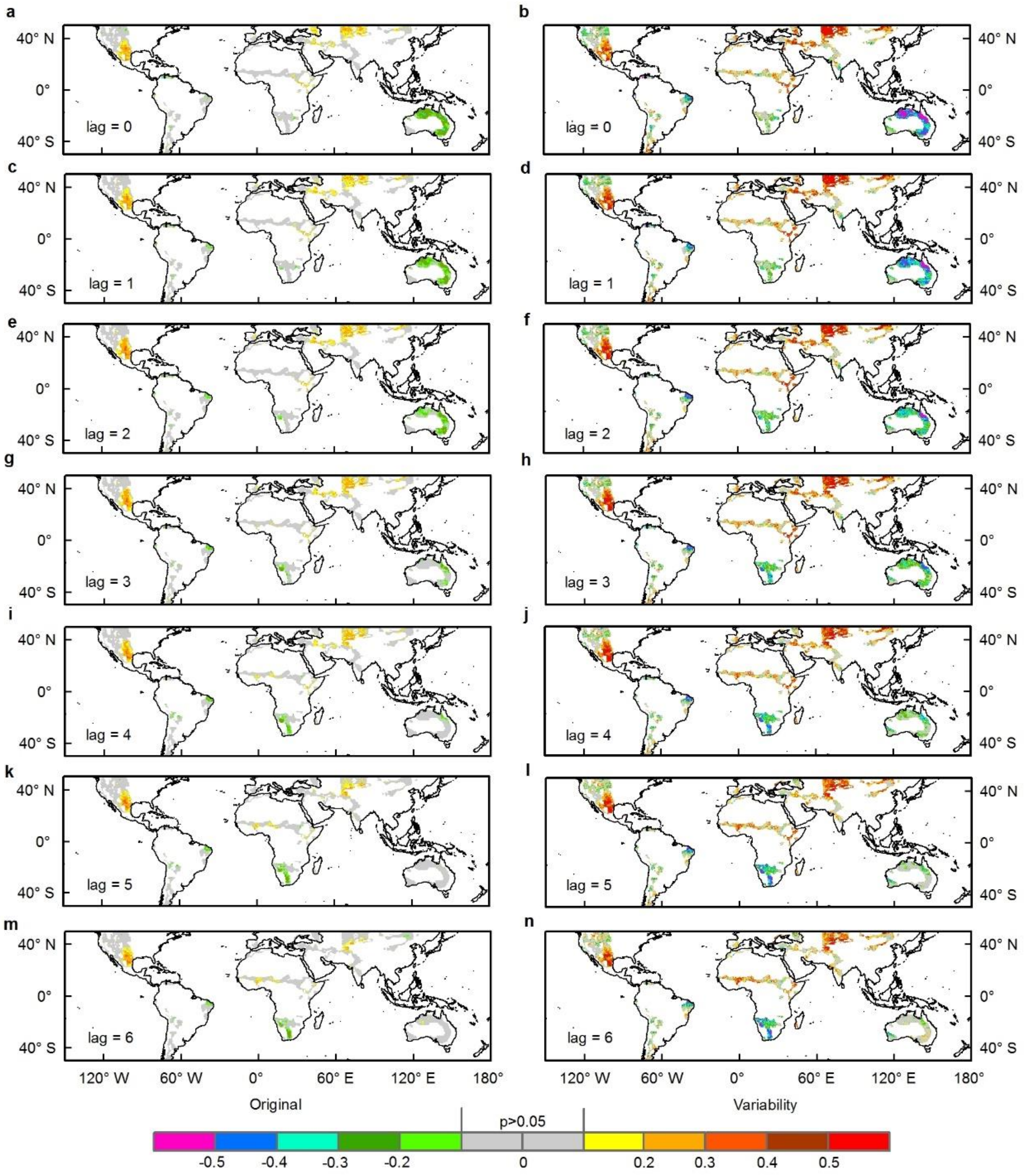

Supplementary Figure 12

Spatial patterns of cross correlations (R) between Niño3.4 index and PCI for Original time-series (a,c,e,g,i,k,m) and EEMD extracted Variability (b,d,f,h,j,l,n) over global semi-arid areas from 2000 to 2014. Lags are indicated in each panel. The maps were created by the ArcMap 10.1 (http://www.esri.com/software/arcgis/arcgis-for-desktop).

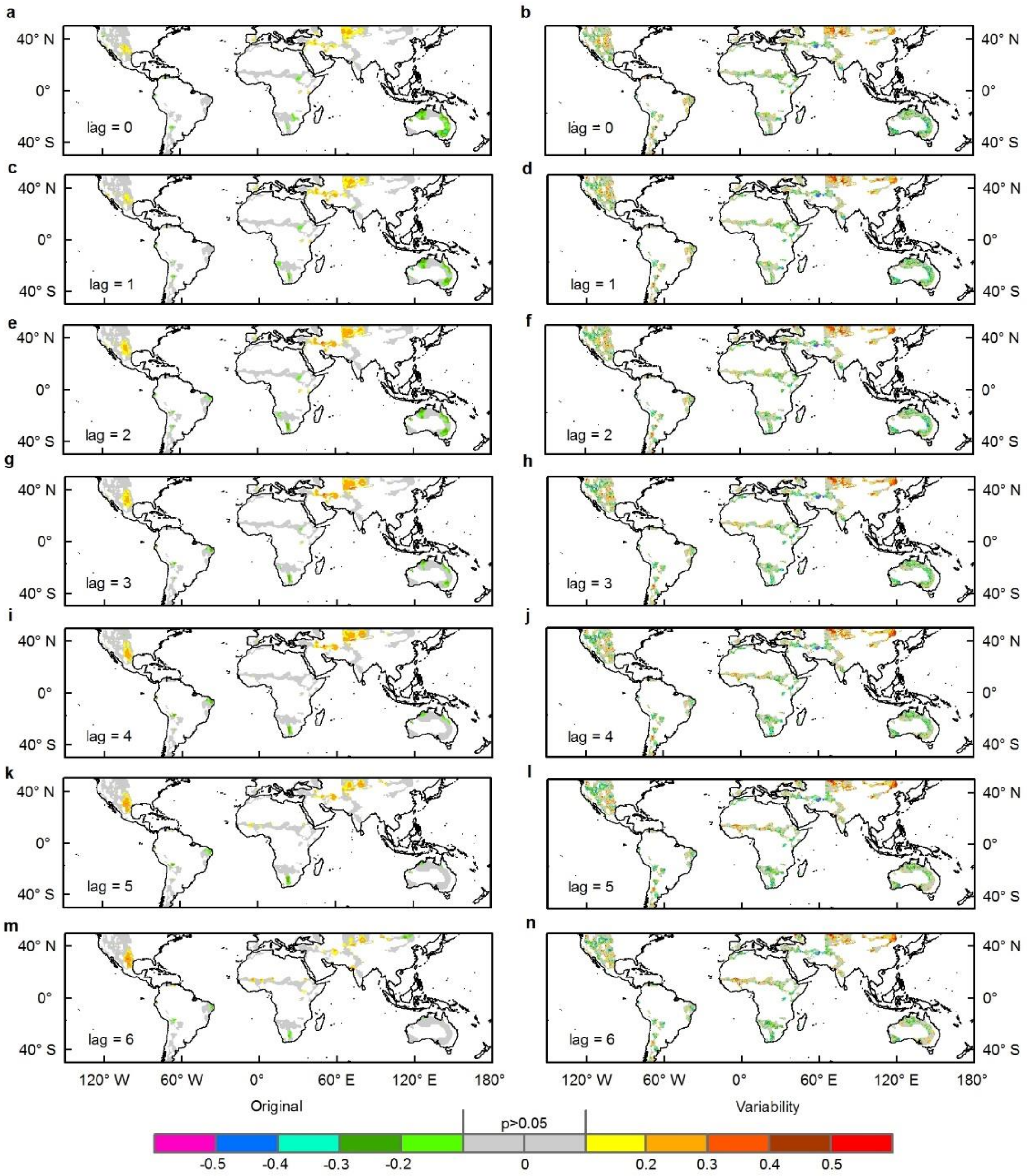

Supplementary Figure 13

Spatial patterns of cross correlations (R) between Niño3.4 index and Climate Research Unit (CRU TS3.23) precipitation anomaly for Original time-series (a,c,e,g,i,k,m) and EEMD extracted Variability (b,d,f,h,j,l,n) over global semi-arid areas from 2000 to 2014. Lags are indicated in each panel. The maps were created by the ArcMap 10.1 (http://www.esri.com/software/arcgis/arcgis-for-desktop).

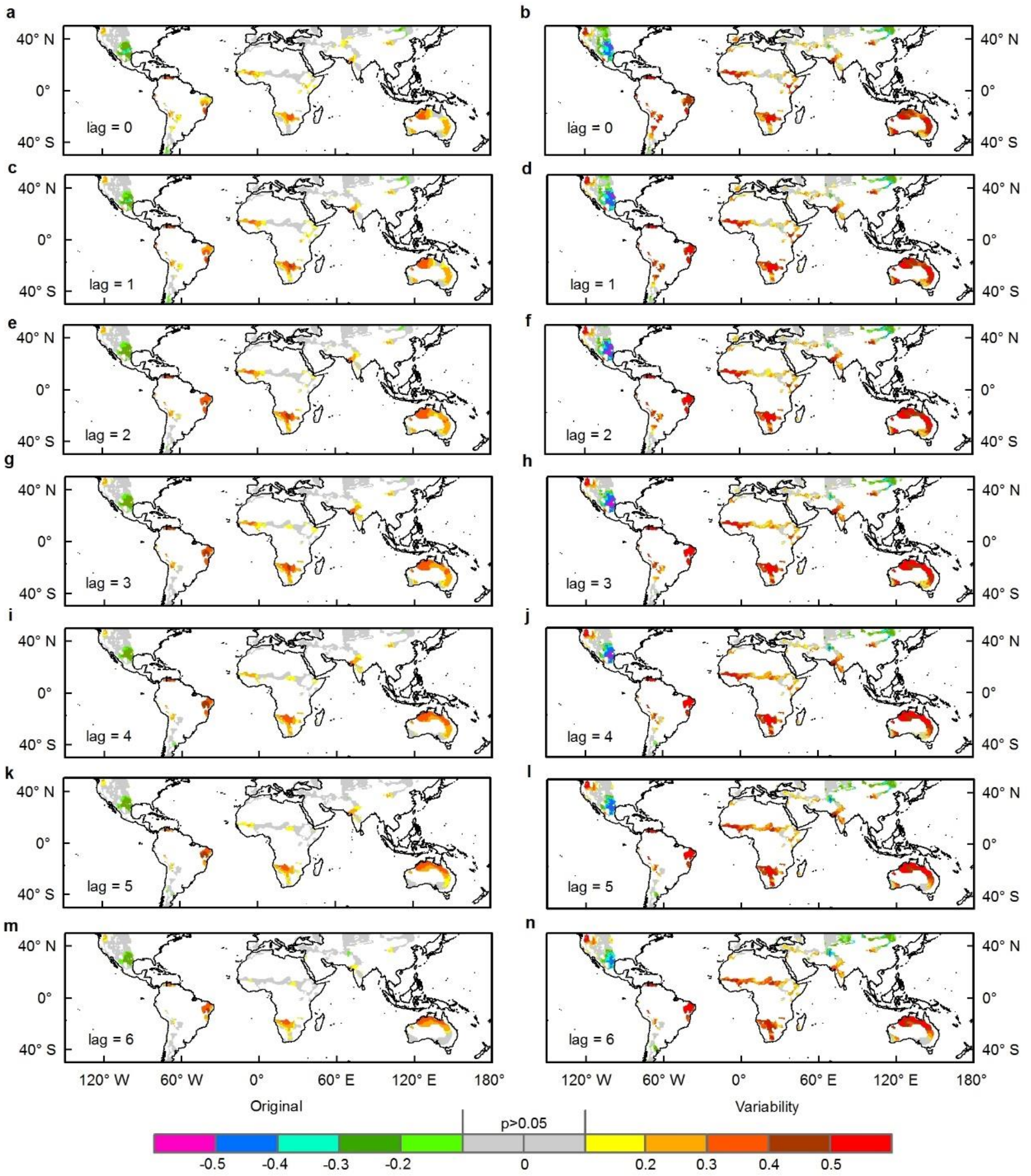

Supplementary Figure 14

Spatial patterns of cross correlations (R) between Niño3.4 index and Climate Research Unit (CRU TS3.23) temperature anomaly for Original time-series (a,c,e,g,i,k,m) and EEMD extracted Variability (b,d,f,h,j,l,n) over global semi-arid areas from 2000 to 2014. Lags are indicated in each panel. The maps were created by the ArcMap 10.1 (http://www.esri.com/software/arcgis/arcgis-for-desktop).

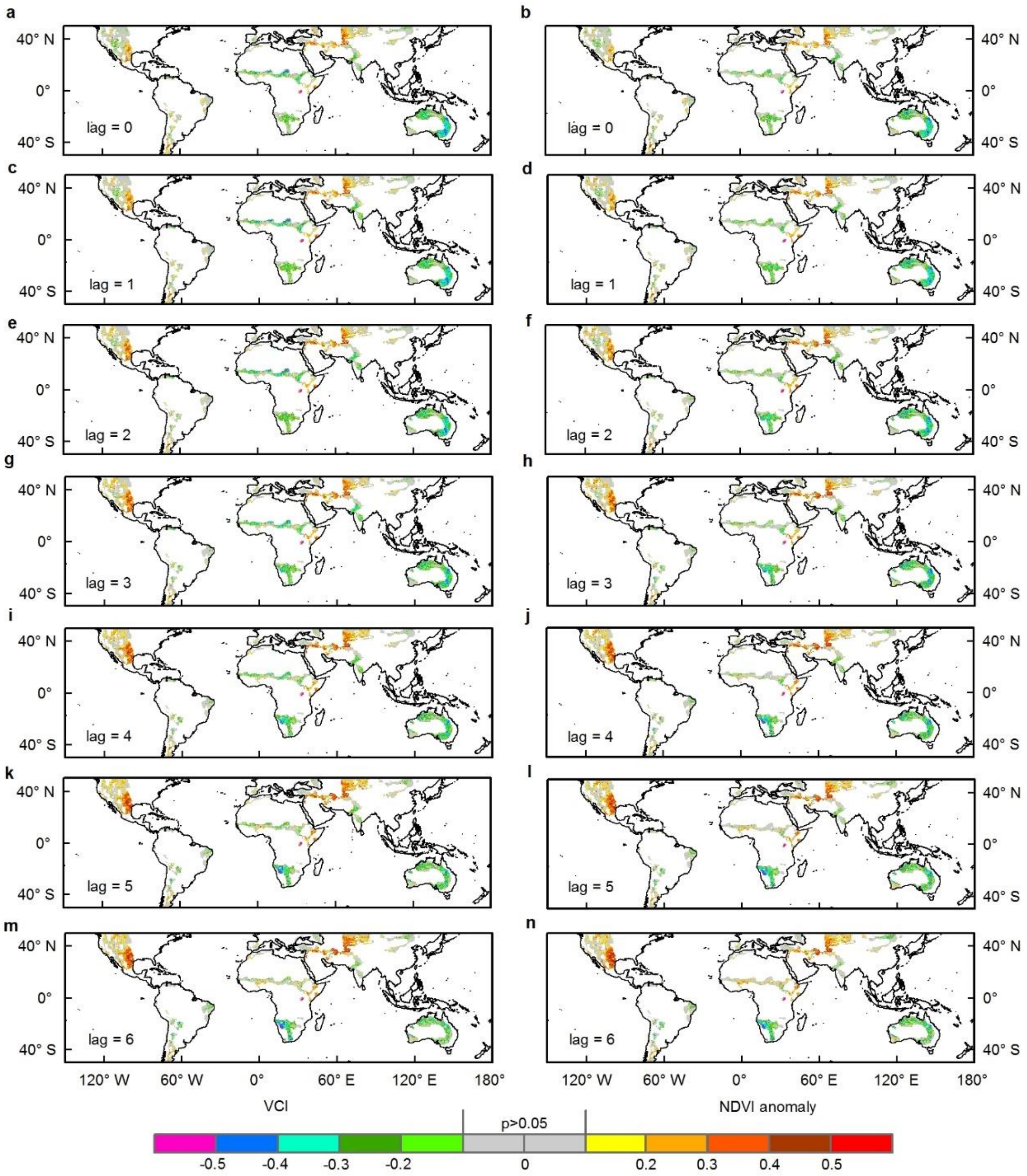

Supplementary Figure 15

Spatial patterns of cross correlations (R) between original time series of Niño3.4 index and MODIS VCI (a,c,e,g,i,k,m), as well as NDVI anomaly (b,d,f,h,j,l,n) over global semi-arid areas from 2000 to 2014. Lags are indicated in each panel. The maps were created by the ArcMap 10.1 (http://www.esri.com/software/arcgis/arcgis-for-desktop).

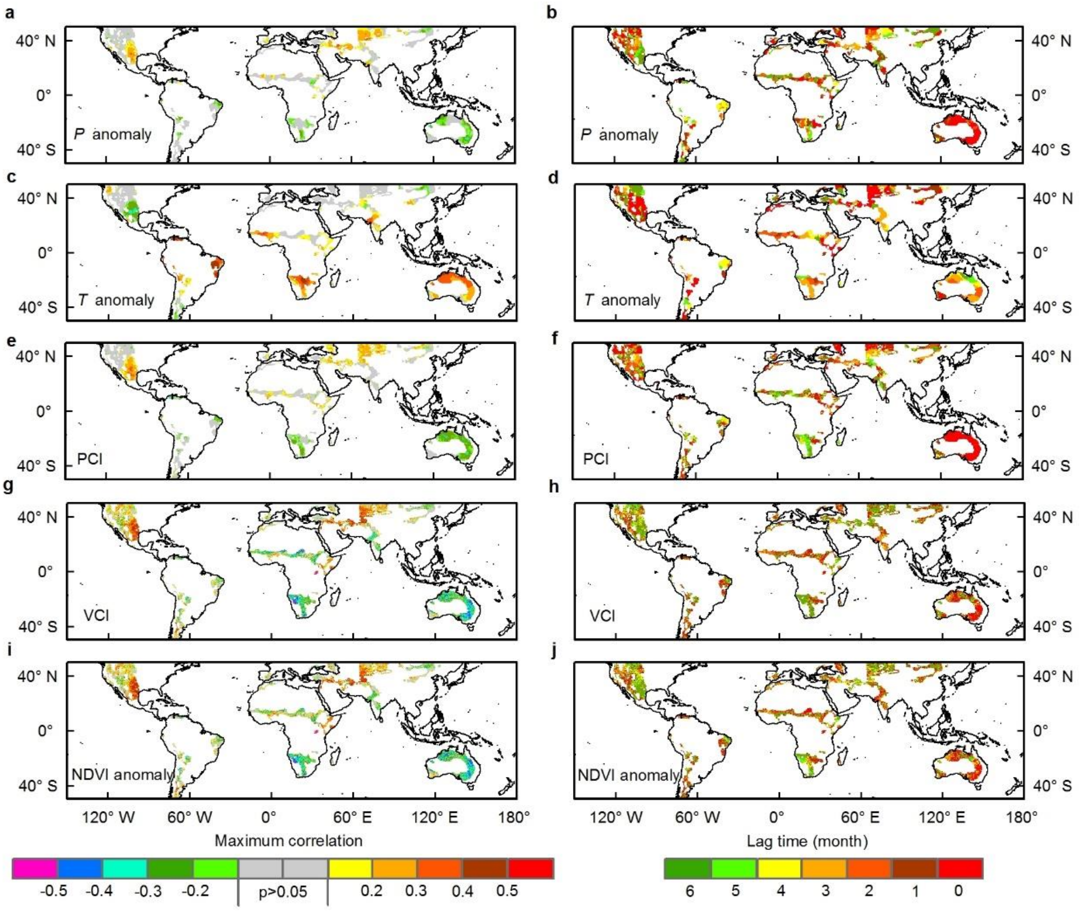

Supplementary Figure 16

Spatial patterns of maximum cross correlations (R) and time lags between original time series of Niño3.4 index and variables over global semi-arid areas from 2000 to 2014. **a**,**c**,**e**,**g**,**i**, The maximum correlations between monthly mean Niño3.4 index and Climate Research Unit (CRU TS3.23) precipitation anomaly (*P* anomaly, **a**), temperature anomaly (*T* anomaly, **c**), TRMM precipitation condition index (PCI, **e**), MODIS vegetation condition index (VCI, **g**) and NDVI anomaly (**i**). **b**,**d**,**f**,**h**,**j**, The corresponding time lags of *P* anomaly (**b**), *T* anomaly (**d**), TRMM PCI (**f**), MODIS VCI (**h**), and NDVI anomaly (**j**) are shown in the right panels. Significance levels (p>0.05) are shown in grey shading. Note that the values are calculated from lag times from 0 to 6 months. Time lags mean variables lagging of Niño3.4. The maps were created by the ArcMap 10.1 (http://www.esri.com/software/arcgis/arcgis-for-desktop).
